# Neutralizing Antibodies and Sin Nombre Virus RNA after Recovery from Hantavirus Cardiopulmonary Syndrome

**DOI:** 10.3201/eid1003.020821

**Published:** 2004-03

**Authors:** Chunyan Ye, Joseph Prescott, Robert Nofchissey, Diane Goade, Brian Hjelle

**Affiliations:** * University of New Mexico School of Medicine, Albuquerque, New Mexico, USA

**Keywords:** hantavirus, Sin Nombre virus, hantavirus cardiopulmonary syndrome, persistence, HCPS, neutralizing antibody

## Abstract

Patients who later have a mild course of hantavirus cardiopulmonary syndrome (HCPS) are more likely to exhibit a high titer of neutralizing antibodies against Sin Nombre virus (SNV), the etiologic agent of HCPS, at the time of hospital admission. Because administering plasma from patients who have recovered from HCPS to those in the early stages of disease may be an advantageous form of passive immunotherapy, we examined the neutralizing antibody titers of 21 patients who had recovered from SNV infection. Even 1,000 days after admission to the hospital, 6 of 10 patients had titers of 800 or higher, with one sample retaining a titer of 3,200 after more than 1,400 days. None of the convalescent-phase serum samples contained detectable viral RNA. These results confirm that patients retain high titers of neutralizing antibodies long after recovery from SNV infection.

Hantaviruses are negative-stranded RNA viruses of the family *Bunyaviridae*. At least 11 members of the rodent-borne genus *Hantavirus* have been associated with hantavirus cardiopulmonary syndrome (HCPS) or hemorrhagic fever with renal syndrome (HFRS) in humans (*1*). Sin Nombre virus (SNV), the prototype etiologic agent of HCPS, is carried by the deer mouse, *Peromyscus maniculatus* (*2*). While four other etiologic viruses cause HCPS in North and Central America and at least two cause HPCS in South America, SNV accounts for most of the >300 known North American cases.

SNV is transmitted primarily by inhalation of contaminated aerosols of rodent urine, feces, or saliva. The first symptoms appear 9–33 days later (*3*). After a prodromal phase of 1 to 6 days, consisting of fever, myalgia, headache, malaise, gastrointestinal disturbances, and thrombocytopenia, hypotension or shock and acute pulmonary edema develop in most patients (*4*). In practice, HCPS is provisionally diagnosed in most patients, and they are admitted to a hospital on the first day that pulmonary edema occurs. In patients with fatal cases, death occurs within 3 days after the onset of respiratory symptoms. Because such a narrow window exists between presentation and lethal outcome, improving the outcome will likely require rapid and decisive intervention, perhaps before the ultimate severity of the disease is known for a particular patient. Since most deaths are caused by myocardial dysfunction and hypoperfusion rather than hypoxia, some investigators have recently begun to use the term hantavirus cardiopulmonary syndrome (HCPS) rather than the previous term hantavirus pulmonary syndrome.

Antibodies of at least the immunoglobulin (Ig) M class are present from the earliest clinical stages of HCPS, and IgG antibodies against either the nucleocapsid (N) or G1 glycoprotein antigen are present in most patients even in the prodrome phase (*5*). Recently, we examined the kinetics of the development of antibodies capable of in vitro neutralization of SNV in patients with HCPS and found that many patients had exceptionally high titers (≥800) of such antibodies from the first day of clinical illness (*6*). In addition, we found that patients who had a milder course of disease had markedly higher titers of neutralizing antibodies on admission than did those patients who later exhibited a more severe infection. Because other acute viral infections have been successfully treated with the plasma of patients who had recovered from these diseases, we are contemplating the use of such treatment for patients with HCPS. Toward this end, we examined the kinetics of the decay of neutralizing antibodies in patients who had recovered from SNV infection 3 months to 5 years before.

## Materials and Methods

### Study Participants

Patients were considered to have acute SNV infection on the basis of the following serologic criteria: the presence of IgM and IgG antibodies directed against the SNV N antigen and the presence of IgG antibodies against the viral G1 antigen. The latter marker is specific for infection with SNV (*7*). A total of 21 samples were collected from 21 patients who were called back for reevaluation as part of a study of the sequelae of HCPS caused by SNV (D. Goade, unpub. data). Informed consent was obtained from patients or their parents or guardians, and human experimentation guidelines of the U.S. Department of Health and Human Services and the University of New Mexico Human Research Review Committee were followed in the conduct of this research.

### Focus Reduction Neutralization Test (FRNT)

The serum samples from HCPS patients were examined by FRNT in at least duplicate analyses in 48-well tissue culture plates (*6*). (We did not subject serum samples to heat inactivation because previous studies had shown that decomplementation did not significantly change the measured FRNT titers of a group of human or rodent serum specimens with titers between 800 and 1,280 [C. Ye, unpub. data].) Samples were serially diluted (1:50, 1:100, 1:200, 1:400, 1:800, 1:3,200, 1:12,800) and mixed with equal volumes of approximately 45 focus-forming units (ffu) of SNV strain SN77734 (*8*) for 1 h at 37°C before incubation on Vero E6 cells. The dilution buffer consisted of complete minimal essential medium (MEM; Gibco/BRL, Grand Island, NY) containing 2.5% fetal bovine serum (HyClone Laboratories, Logan, UT). After adsorption for 1 h at 37°, the cells were washed in phosphate-buffered saline (PBS) and overlaid with medium containing 1.2% methylcellulose for 7 days. The methylcellulose layer was removed, and the cells were fixed with 100% methanol with 0.5% hydrogen peroxide. Viral antigen was visualized by the addition of hyperimmune rabbit anti-SNV N protein (1:5,000), followed by peroxidase conjugated goat anti-rabbit IgG (Jackson Immuno Research Laboratory, West Grove, PA) and DAB/metal concentrate as substrate (Pierce Chemicals, Rockford, IL). The neutralization activity of a patient’s serum specimen was expressed as the maximum serum dilution that would reduce the number of viral foci by 80% or more.

### Reverse Transcription–Polymerase Chain Reaction (RT-PCR)

RT-PCR was conducted as described previously (*8*). The nested protocol in question has a sensitivity of <10 copies of viral RNA per reaction when RNA is isolated from cell-free materials such as serum (J. Botten, B. Hjelle, unpub. data). The primers, which have been described previously (*8*), were located near the 5′ terminus of the gene encoding the SNV N antigen. Many sequences spanning this region have been examined from SNV isolates from throughout the United States and Canada, and little variation has been detected (B. Hjelle, unpub. data). In each case, RNA was prepared from 100 μL of serum and 20% of the preparation was subjected to nested RT-PCR analysis.

## Results

The Table shows the geographic origin, age range, sex, and clinical score of each of the 21 patients. Patients traveled to Albuquerque, New Mexico, as funds permitted from the University of New Mexico’s Research Allocation Committee. The patients were subjected to a battery of clinical tests, including studies examining pulmonary, renal, and hepatic function. The results of the clinical studies will be reported separately.

**Table:** Demographic and clinical characteristics of 21 patients with history of Sin Nombre virus infection^a^

Patient	Age at time of illness	Sex	Date admitted	Day serum collected	Location	Severity^b^
1	10	M	30 Mar 1999	589	NM	I
2	44	F	4 Dec 1995	1789	NM	IIE
3	28	F	5 Apr 1999	592	NM	0
4	28	F	3 Apr 1999	566	WA	II
5	42	M	15 Jun 1999	94	NM	I
6	29	F	1 May 1998	1063	NM	I
7	18	F	11 Aug 1998	512	CO	IIE
8	44	F	29 Mar 1994	1627	NM	II
9	43	M	1 Sep 2000	231	AZ	IIE
10	41	M	20 Mar 1997	1436	KS	II
11	39	F	6 Mar 2001	85	NM	II
12	50	M	20 Aug 1998	334	NM	IIE
13	34	F	15 Jul 1999	92	CO	I
14	44	F	3 Nov 1997	1281	KS	II
15	19	F	18 Oct 2000	132	MT	II
16	27	M	23 Jul 1997	1356	NM	I
17	27	F	27 Jan 1998	1196	KS	I
18	23	F	30 Oct 1999	161	NM	IIE
19	16	M	19 Sep 1997	1419	TX	II NO (*15*)
20	21	F	27 May 1994	2268	NM	II
21	44	M	20 Dec 1997	1056	WI	II

^a^F, female; M, male; NM, New Mexico, WA, Washington, CO, Colorado; AZ, Arizona; KS, Kansas; MT, Montana; TX, Texas; WI, Wisconsin. ^b^Severity scale: Class 0, prodromal symptoms with seroconversion but with no cardiopulmonary manifestations; Class I, prodrome symptoms with hypoxia but not requiring endotracheal intubation; Class II, survived but required endotracheal intubation; Class IIE, required extracorporeal membrane oxygenation; II NO, treated with inhalational nitric oxide. Because they required endotracheal intubation, patients in Class II are regarded as having been more severely ill than those in Classes 0 and I.

Serum samples were collected for virologic studies and examined for residual viral RNA by RT-PCR and for neutralizing antibodies. Although positive control RNA preparations produced amplification products as expected, no viral RNA was detected in any of the patients’ serum samples (data not shown). However, the serum samples examined continued to exhibit neutralizing antibodies at titers ranging from 100 to 3,200 (Figure). No statistically significant relationship existed between neutralizing antibody titers after the patient’s recovery from SNV infection and the severity of the previous illness, nor did a relationship exist between antibody titers and the use of extracorporeal membrane oxygenation (ECMO) in our patient group.

**Figure:**
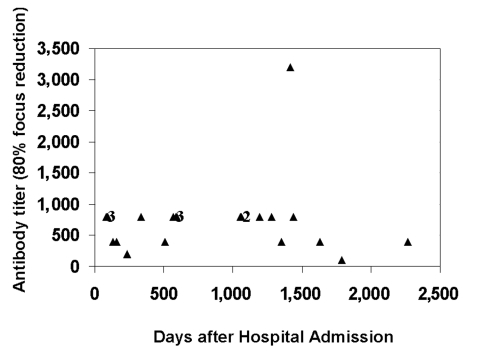
Titers of neutralizing antibodies against Sin Nombre virus (SNV) strain SN77734 in serum samples from patients surviving hantavirus cardiopulmonary syndrome due to SNV. The reciprocal of the endpoint neutralization titer is plotted for each sample. Numbers near certain clusters of points reflect the number of individual data points represented in a particular cluster.

## Discussion

Although case-fatality ratios for HCPS have declined since the initial description of the disease, probably reflecting improved recognition of mild illness, therapeutic options remain limited. The efficacy, if any, of antiviral drugs such as ribavirin remains to be demonstrated. Intensive care management of the manifestations of the disease constitutes the primary method of clinical intervention. At some centers, ECMO has been utilized in those patients who exhibit hemodynamic or pulmonary instability that is predictive of fatal outcome (*9*). (Six of our patients underwent this procedure.)

Although treating patients with viral hemorrhagic fevers with the plasma of patients who have recovered from the disease is often proposed or discussed, rigorous demonstration of its efficacy in humans or animals is often lacking. Argentine hemorrhagic fever (AHF) due to Junín virus and other arenaviral diseases are among the exceptions (*10*–*13*). The efficacy of convalescent-phase plasma in treating AHF was demonstrated before the nature of the protective substance contained therein was positively identified. With the advent of a test for neutralizing activity, Argentine investigators retrospectively demonstrated a correlation between the quantity of the neutralizing activity of plasma and the degree of protection afforded by its infusion (*11*).

The neutralizing activity of a dose of plasma is defined by multiplying the reciprocal of the highest dilution that would reduce the number of viral foci by 80% by the volume of the inoculum, in milliliters. Overall, the infusion of convalescent-phase plasma containing antibodies to Junín virus reduced deaths by approximately 90%. In a retrospective analysis, investigators showed that those patients who received >2,000 U/kg of neutralizing antibodies appeared to respond better than did those patients who received 1,000–2,000 U (*11*).

Because HCPS is rare, the number of treatments that can be independently examined through clinical trials without delaying the identification of an effective intervention is inherently limited. To determine whether a clinical rationale exists for attempting passive immunotherapy for HCPS with neutralizing antibodies, we first studied the relationship between the titer of such antibodies on a patients’ admission to the hospital and the clinical outcome. That study showed a profound and inverse relationship between antibody titer and disease severity (*6*).

It then became important to determine whether the small number of patients who have recovered from SNV infection might constitute a pool of plasma donors of sufficient size to provide a stable source of plasma to treat future patients. The study reported herein was initiated to determine whether neutralizing antibodies persist for substantial periods after recovery from the disease. Results indicate that the answer is affirmative but that not all recovered patients are equally rich sources of neutralizing antibodies.

Modern techniques for collecting plasma allow for convenient and low-risk preparation of 500–600 mL or more of plasma from healthy donors; this can be repeated on multiple occasions over several weeks. For that and other reasons, each of those patients whose titers remained at >800 represents a rather rich source of antibodies. If one extrapolates from the experience of the Argentine investigators in their treatment of AHF, a single 600-mL plasma donation by a person with a neutralizing antibody titer of 800 can be used to treat two 80-kg (~175-lb) patients. A patient such as the person whose titer was 3,200 could be identified as a particularly valuable repeat donor, one whose every donation could be divided and used to treat eight new patients with HCPS.

Thus far, little or no overlap occurs among the behavioral characteristics associated with HCPS and those associated with infection by bloodborne pathogens such as HIV or hepatitis C virus. Potential donors would be subjected to the same battery of questions and tests for bloodborne pathogens as would any other directed blood donors, with the exception that donors would be allowed to donate despite the history of hospitalization for HCPS and receipt of blood products during ECMO therapy. Although we therefore expect that most potential donations of convalescent-phase plasma would be free of bloodborne pathogens, ensuring that SNV has been completely cleared from the blood is more difficult. Thus far, no evidence for a persistent state of SNV infection in humans has been shown. In fact, studies published to date show that in all cases that could be evaluated, SNV is undergoing rapid clearance at the time of clinical presentation (*14*).

Although our RT-PCR data are reassuringly negative, we cannot know whether a larger dose of convalescent-phase plasma would contain infectious SNV. In fact, SNV has never been cultivated from any human source, despite attempts by multiple laboratories.

We believe that passive immunotherapy may be efficaceous if administered very early in the course of illness, before the severity of a particular patient’s disease course is known. To reduce the risk for accidental inoculation of potential recipients of anti-SNV convalescent-phase plasma, at least two strategies are possible. First, one could impose a delay of 6 to 36 hours by requiring that potential recipients have a positive specific test for SNV antibodies before the plasma is administered. Alternatively, one could impose either purification or viral inactivation procedures for the antibodies or await the development of human or humanized monoclonal antibodies before instituting this therapy. We presume in our risk analysis that no new risk is associated with the inadvertent inoculation of a second strain of SNV in a patient who is undergoing active infection with a different genotype of the same virus, since there is no a priori evidence to suggest otherwise.

No current evidence suggests that infusing anti-SNV antibodies would enhance the pathogenesis of SNV. The data of Bharadwaj et al. (*6*), in fact, support the presumption that treatments that lead to higher titers of neutralizing and even nonneutralizing anti-SNV antibodies are more likely to lead to less severe disease. We believe that convalescent-phase plasma therapy may represent a practical intervention that is feasible, given current resources and funding levels for potential antihantavirus therapies.
